# Threshold Concepts in a Literary Magazine Produced by Health Care Students

**DOI:** 10.15694/mep.2020.000007.1

**Published:** 2020-01-08

**Authors:** Johanna Meyer, Hanna Chang, Virginia Randall

**Affiliations:** 1 Uniformed Services University of the Health Sciences

**Keywords:** threshold concepts, literary magazine produced and edited by medical students, thematic qualitative analysis, medical student authors

## Abstract

This article was migrated. The article was marked as recommended.

**Introduction:** *Progress Notes* is a literary magazine featuring works from health care students in the USA. It is edited by medical students at the Uniformed Services University of the Health Sciences (USUHS) to provide a space for their creative works. Since the magazine’s inception in 2016, four volumes have been published, containing 130 works of poetry, fiction, reflection, and artwork.

**Goal:** To understand the themes and perspectives of the published works.

**Methods:** After Institutional Review Board approval, two researchers used qualitative thematic analysis to examine the texts, coding independently and resolving differences by discussion. They arranged the codes into themes which were discussed until consensus. A third researcher read the texts, codes and themes and verified that they were an accurate reflection of the published works. Artwork was assigned themes by the same two researchers who analyzed the written works, and a sample selection was verified by the third researcher.

**Results:** Researchers identified eight themes across poetry, fiction, and reflective essays: vocation, death, failure/resilience, emotional restraint, personhood of the patient, approach to the patient, military physicians, and moments of personal realization. Four themes were identified in the artwork: death; comradeship/aloneness; vocation/quest; and competence.

**Discussion:** Students submitted creative works in which they grapple with what it means to be a physician. Analyzed through the lens of the threshold concepts, researchers identified: “I am a healer;” “I can deal with ambiguity;” “The patient is the focus;” “As a military medical officer, I serve two masters;” and “As a physician, I have a unique and complex relationship with death.” These threshold concepts represent an ontological shift in the students’ professional identity.

**Conclusion:** A literary magazine edited, and featuring works by, health care students provides a forum in which health care students wrestle with the elusive and enigmatic fundamental principles of being a physician.

## Introduction

There is a growing realization within the community of medical educators that there is more to becoming a healthcare provider than mere rote memorization, despite the ever-increase amount of medical information and knowledge to be mastered. The theory of threshold concepts suggests that there are certain fundamental principles, apart from strict academic knowledge, that the student must come to appreciate during their professional development, and it is only after the student has made these realizations that they are able to view the world through the lens of their profession.

The creation of a new professional identity implies, at worst, destruction of the old personal identity and, at best, its integration into the new. However, this process requires space for the student to reflect on these experiences, which often cause them to question what it means to be a physician, and, hopefully, to come to some resolution.

*Progress Notes* is a peer-reviewed literary magazine featuring original works by medical students from USUHS and Health Professions Scholarship Program (HPSP) programs. The journal is edited by medical students at USUHS. Each year, students in these programs, from across the country, submit pieces reflecting upon their experiences in medical school, and of the human condition generally, through art, poetry, fiction and reflective essays. Since its inception in 2016, the journal has received a total of 249 submissions, of which 130 were published.Authors were predominantly third- or fourth-year medical students from USUHS and HPSP. However, as noted above, the call for submissions was open to all federal healthcare students and pieces were published from dental and nursing students, a 5 ^th^ year MD/PhD student, and a clinical psychology student.

The editing staff consisted of third- and fourth-year USUHS students, each paired with a USUHS faculty member. Pieces were accepted primarily based on their literary merit and emotional impact; however, their interaction with the other works featured within the journal was also considered. Editing included clarifying discussions between the student editors and the student authors.

It is our assertion that this magazine has created a space in which students are able to grapple with the critical insights and threshold concepts that are essential to becoming a physician. During the thematic analysis of the four previous editions of *Progress Notes*, it became apparent that the creative pieces centered around the students’ conception of their vocation to become a physician and what that process entailed.

## Methods

Research Goal

To describe and understand the themes and perspectives of the works submitted by health care students.

Methods

With IRB approval, we used qualitative thematic analysis to examine 130 reflective essays, stories, poems, and artworks published in *Progress Notes* (volumes 1-4, 2016-2019). Two investigators (JM, VR) performed the initial analysis by reading and rereading the written pieces from the first three volumes, independently coding them line-by-line. Using constant comparison, we placed each phrase within an existing code or created a new code. We were careful to use the authors’ own words rather than interjecting our interpretations. We discussed each code and phrase until consensus. We then independently arranged the codes into themes and sub-themes, which we then discussed until we reached consensus. Using this thematic construct, we coded the fourth volume directly into the agreed upon themes, to assure ourselves that the themes could adequately accommodate all the codes and phrases.

We next discussed the themes and sub-themes until we had a thorough understanding of their breath and scope and then formatted them as an exhaustive list of the ideas represented in the pieces. We collapsed this list into eight themes, each with sub-themes, definitions, and illustrative quotes. For a portion of the thematic analysis, the software NVivo was used to correlate quotes and codes.

A third investigator (HC) independently reviewed the coding to crosscheck the accuracy of the coding in reflecting the authors’ intended content. As a group, we then discussed the coding and themes until consensus.

In instances in which it was difficult to pinpoint the authors’ intent, we reviewed their self-described biography and whether the piece was submitted as fiction or reflective essay, to assist in coding. Despite these external clues, there is inevitable bias, as we relied on our own experiences and intuition when unsure how to code a passage of written text.

For the artwork, because we could find no template for qualitative analysis of art (as opposed to artistic analysis), we independently assigned a theme to each piece of art, then discussed the themes until consensus. Remarkably, the researchers found common language surrounding this artwork and this was verified through triangulation with an independent researcher using a sample of the published artworks.

We then examined the final statement of themes, to determine if any rose to the level of a threshold concept (specifically: had the characteristics of ontological shift in understanding of who the student felt themselves to be; involved a troublesome (liminal) phase; and was consistent with our own experiences and understanding of threshold learning experiences during medical school and a medical career).

One of the authors (JM), a 4 ^th^ year medical student (class 2020), obtained her B.A. and M.A. in Comparative Literature. Her insight into the creative process of writing and art informed our analysis, discussion and conclusion.

## Results/Analysis

Over four publications, 249 works were submitted and 130 were accepted and published, consisting of 10 works of fiction, 40 poems, 21 reflective essays and 59 pieces of visual art. Using thematic analysis, eight themes were identified in the written works:

***Vocation***: A vast majority of the authors saw being a doctor as more than a skill or trade to be learned, but rather as a personal vocation. Thus, the process of going through medical school was frequently described using imagery of a quest, during which the authors struggled to recast themselves as physicians, similar to Campbell’s Hero’s Journey ( Campbell, 1988) with legitimate acceptance into the profession as the ultimate reward.

“How blessed we are, how sacred, to repair these human beings.”

“...the attending turned my way and gave me a firm handshake and a knowing smile. Perhaps his way, I imagined, of welcoming to the club”

***Realization** **:*** The authors realized that their original understanding of themselves, the situation, or the patient was fundamentally incorrect, and they reinterpreted their experiences through a new perspective. Some of these realizations are learning thresholds or threshold experiences.

“Engaging with death instead of attempting to prolong life was a new challenge.”

***Failure/Resilience*:** In a number of the selections, the authors vacillate between overconfidence and despair at their incompetence. These personal assessments are tied to their feelings of self-worth, and demonstrate how the students’ personal identity becomes inextricably entangled with their professional identity. This personal damnation is also tied to the theme of isolation, in that the students each feelthat they are unique in their failure.

“Then the voices began to call out: voices of doubt, failure and incompetence. My hidden fears, previously kept at bay by the light, were given full voice here. My inability to resist the whispers terrified me as I drowned in nothingness.”

“I used to think I was smart. Now I just know that I’m better at faking it.”

***Emotional Restraint:*** The student authors frequently discussed the process of learning the unspoken rules of the medical profession. In particular, a strong theme throughout was the belief of the authors that they should, now, hide their emotional reactions behind a mask of professional composure.

“A gentle hand sobers my primal noise

‘Doctor, your next patient is in the O.R.’

I choke back one last salty drop

Knowing that I am completely human

and trying to prove that I’m not.”

***Death*:** How one should engage with death was a prominent theme throughout. Is it a natural part of life, to be accepted and prepared for? Is it to be feared? Is death an adversary against which a physician battles, to either triumph or fail? Or is the ultimate tragedy that we all die alone in the end? There was often more poignant sadness at this state of isolation than at the prospect of death itself.

“He remained my patient until he was nobody’s patient - a few days later when he passed away.”

“[Death] Stole away the smallest souls, undeterred by how hard we tried”

***Personhood of the patient*:** Many of the pieces described how the humanity of the patient was forgotten, forced into the background by the process of acculturation in medical school. However, when the authors interact directly with the patients, in their vulnerable states, they come to recognize how their programed responses created distance between themselves and their patients, and through that recognition to rediscover their patients’ humanity.

“For hours I studied, put in the time, pored over texts, thought I’d be fine, then entered the room, finally seeing, I’d unlearned how to engage human beings.”

***Approach to patient*:** Several authors identified various ways in which they had distanced themselves from the patient either by objectifying the patient or treating the patient as a means to an end, instead of being concerned with the well-being of the patient as a good in and of itself. Patients became diagnoses, or check boxes in an algorithm, or opportunities to impress an attending, or learning opportunities, rather than individuals looking for help.

“I realize how easy it had become to insulate myself with evidence-based practice and empiric treatment.”

***Military Physicians*** **:** Both military camaraderie and medical collegiality are incredibly strong bonds. Military physicians bridge these two communities. Even as they profess to be entirely a member of each profession, there is always a pull in the other direction, with the result that only other military physicians can truly understand this tension and thus provide the comfort and support needed to maintain this balancing act.

“Sense and duty they collide, What to do? How to decide?”

## Discussion

Intent

The intent of this research was to understand the themes and perspectives of student healthcare providers using qualitative analysis to examine the compositions, both written and visual, submitted by medical students and published in four volumes of *Progress Notes.* It is important to stress that we are not making claims as to the essential nature of medicine. Rather, we are seeking a better understanding of the students’ perception of the medical profession, in the hope that such an understanding will aid in improving medical education

Masks

The students often described the process of learning the unspoken rules of the medical profession. In particular, many authors referred to the unexpressed expectation that they should, now, hide their emotional reactions behind a mask of professional composure. Several of the visual pieces were of papier-maché masks, which have the inherent property of hiding the true expression (or nature) of the wearer.

One student remarked upon the magical effect his attending’s smile had on her patients and wonders “how many times she’s rehearsed it” and wishes that they could have a class on it next year. This smile that acts as a mask to hide the inner thoughts of the attending was mirrored in another piece, where a third-year student noted that her attending’s smile “[d]oesn’t reach his eyes.” Even as the attending praises her work, the student notes that “his eyes say nothing.” However, while the student finds this emotional disconnect disconcerting, by the end of her clerkship year, she finds herself expressing canned sympathy for a patient and notes that her own smile “doesn’t reach [her] eyes.”

The student authors appear to be simultaneously trying to emulate the professionals around them and question the type of professionals they wish to become. In this process, they are challenging the often unstated, or hidden, curriculum of professional identity formation. As one author asserts, “[s]omeday, I know, I’ll smile just like they do, but right now I’m not sure that’s a good thing.”

Creating distance from patients

Several authors identified various ways in which they had distanced themselves from their patients either by objectifying them or by treating the patient as a means to an end, instead of being concerned with the well-being of the patient as a good in and of itself. Patients became diagnoses, or check boxes in an algorithm, or opportunities to impress an attending, or learning opportunities, rather than individuals looking for help. Indeed, as the authors found themselves stepping deeper into the world of medicine and becoming acclimatized, the clinical experiences they chose to focus on were moments when they found themselves, or their mentors, forgetting the humanity of the patient.

It was only through direct interaction with patients, watching a family member become sick, or being a patient themselves, that the authors were able to recognize how their programed responses created distance between themselves and their patients. Through that recognition they were able to rediscover their patients’ humanity.

Threshold Concepts

However, almost all of the pieces ended with a moment of realization on the part of the author, where they admitted that their perception of the situation, themselves or the patient had drastically changed. They reinterpreted their experiences through a new perspective. When viewed through Meyer and Land’s ( Meyer and Land, 2005) theory of threshold concepts, some of these realizations can be identified as threshold concepts or experiences.

Threshold concepts is a theoretical framework originally proposed by Meyer and Land to describe how an individual’s perceptions and reasoning are transformed through the process of learning to be a member of a given profession. This framework has been used to examine the educational structure of multiple professions and to identify definitive concepts, whose grasp separates members of that profession from the layperson far more than any intellectual understanding of abstract knowledge. These concepts have key and definitional characteristics. First and foremost, they are *transformative*, representing an ontological shift in the student’s perception. They are *integrative,* in that they allow the student to bring what were originally seemingly disparate, unrelated or troublesome experiences or pieces of knowledge together into an integrative whole. However, they are also *troublesome* in that these concepts are not intuitively grasped, but are only truly appreciated by the student experiencing the training of that profession first hand. The final definitive characteristic of these threshold concepts is that they are *irreversible* and that once grasped, the newly minted professional cannot return to their old way of seeing the world.

While the first three criteria were easily seen in the moments of realization identified in the pieces, the final characteristic, irreversibility, was more elusive, given that we are analyzing a single snapshot in the larger arc of these authors’ professional identity formation. It is difficult to say without longitudinal analysis that these realizations are irreversible and not a way point in the ‘toing and froing’ inherent in a threshold concept.

I am a healer

The moments of realization identified in *Progress Notes* centered around five crucial ideas, which were found to meet the criteria of threshold concepts. The first was the assertion that, “I am a healer,” when the author no longer identifies themselves primarily as a student, but takes ownership of the responsibility of caring for the health of another person. This can be seen as the moment when the student “move[s] from ‘legitimate peripheral participation’ in medicine’s community of practice to full participation” ( Cruess, Cruess and Steinhart, 2019).

In accepting the mantle of ‘I am a healer,” the student is turning the focus away from the patient as an opportunity to learn or to impress their attending and instead striving for the patient’s individual well-being.

Patient-first approach

This leads to another threshold concept where the patient is the focus of the medical interaction. While the ‘patient-first’ approach is something of a tired trope in modern Western medicine, it is very different when a student truly incorporates this principle into their professional outlook.

Struggle with ambiguity

Ambiguity was another difficult concept for several of the authors. There was a struggle and a desire for a definitive answer or diagnosis. Initially, many authors were frustrated by their attendings, who would go forward in their management plans without a definitive diagnosis, and yet eventually, there was recognition that the ability to handle this ambiguity was essential to becoming a doctor.

Instead of searching for the ‘right answer,’ students became comfortable with holding multiple possibilities simultaneously in their heads. As more and more information is learned and incorporated into the decision-making process, these possibilities shift in likelihood and affect the next steps in management.

Military physician

Military physicians serve two, sometimes competing, masters. They develop a unique professional identity.

Death

The final threshold concept identified was the recognition that the physician’s relationship with death was fundamentally different from that of the layman. While this may in large part depend on one’s specialty, physicians often occupy a space where they are perpetually interacting with death, either fighting it or helping their patients’ transition and accept it on their own terms.

Transformation

While the process of grappling with these threshold concepts is troublesome and essentially involves much “toing and froing” on the part of the student, at some point in the development of the professional identity of the individual they are definitively grasped and become “invisibly integrated into [the] professional and personal identity [of that individual]” ( Randall, 2018).

This transformation has great significance, and indeed, is a central purpose of medical school, but is entirely separate from any degree conferred by an institution. However, there has been a growing consensus among medical educators that professional identity formation should play a role in the didactic side of the medical curriculum and even be an “institutionally supported, intentionally crafted, and longitudinally integrated [part of the] curriculum across all years of medical school” (Chandran *et al.,* 2018). Often, this has taken place through the use of preceptor-facilitated small group discussion and reflective essays.

Progress Notes

*Progress Notes* provides a space for “protected venting, and guidance for reflection” ( Egnew *et al*., 2018). A literary magazine also allows students to see that they are not alone in the liminal space. The fear that rings true again and again is the assertion, “I assumed something must be wrong with me.” Many students are, in fact, shocked that they are not alone in these feelings of isolation, incompetence and self-doubt.

Consequently, we would submit that a literary magazine, far from being a frivolous extracurricular activity, is, in fact, an essential part of the underlying curriculum of the school. It allows students to digest and grapple with the hidden curriculum.

Limitations

Limitations to generalizability include that the majority of authors and all the editors are students at the only military medical school in the country. Thus, the themes identified could be limited to this unique population.

However, a significant sample size was analyzed. The volumes were edited by students and students participated in the research as co-investigators, creating face validity. The results were verified by an independent researcher.

## Conclusion

The investigators began this thematic analysis with the goal of gaining insight into the thoughts and feelings of trainees during their health care education. In our analysis, we noted the candidness with which the students freely explored their emotional reactions. *Progress Notes* provided the forum for the students to capture their changing perspectives about themselves as they navigated their journey toward becoming a professional. The prevailing themes expressed in *Progress Notes* were: medicine as a vocation, moments of realization, personhood of the patient, approach to the patient, failure/resilience, emotional restraint, death and military physician-hood.

Through our analysis, we were able to get a snapshot of the students’ experience as they faced different threshold concepts important to their professional identity formation. Threshold concepts of “I am a healer,” “I can deal with ambiguity,” “As a military medical officer, I serve two masters,” “Patient is the focus,” and “Physicians have a unique and complex relationship with death” were consistently noted throughout our review.

A benefit to using a literary magazine format is that these threshold concepts resonated with the medical student editors and as part of the editing process, multiple, fruitful discussions about the works were exchanged between the editors and the creators of those works. Consequently, the feeling of isolation and personal failings of the authors and artists could be normalized through these exchanges. The peer-review component of *Progress Notes* provides an avenue to further the discussion about their experiences of professional identity formation amongst the student population.

## Take Home Messages

• A medical school literary magazine, far from being a frivolous extracurricular activity, is, in fact, a needed part of the underlying curriculum of the school, because it allows students to digest and grapple with what it means to a physician.
• The lack of format, subject matter prompts, and grade allows for a more open and candid conversation.
• Using the lens of thresholds concepts allowed us to see that some of the themes met the criteria for threshold concepts, in that they were transformative, integrative and troublesome.
• A literary magazine allows students to westle with feelings of failure and isolation.

## Notes On Contributors

Dr. Virginia Randall (ORCID: https://orcid.org/0000-0003-2944-9015) is a pediatrician at the Uniformed Services University (USU). She was an active duty pediatrician in the US Army for 30 years before moving to USU where she is interested in medical student education and always involves medical students in her research as co-investigators.

Ms. Johanna Meyer is an ENS in the US Navy and holds a BA and MA in Comparative Literature. She is pursuing a residency in internal medicine.

Ms. Hanna Chang is a 2nd Lt in the US Air Force and holds a BA in Chemistry. She is pursing a residency in pathology.
