# Supplementary material for: Threshold Concepts in a Literary Magazine Produced by Health Care Students
Source: MedEdPublish (2016). 2020 Jan 8;9:7. [Version 1] doi: 10.15694/mep.2020.000007.1 (PMC10697432; doi:10.15694/mep.2020.000007.1)
Supplement: Peer review history

# Reviewer report: Reviewer response for version 1

**DOI:** 10.21956/mep.19957.r30320

**Published:** 2020-04-21

**Authors:** Ken Masters

**Affiliations:** 1 Sultan Qaboos University

This review has been migrated. The reviewer awarded 4 stars out of 5 An interesting article on threshold concepts in a literary magazine produced by health care students. The paper is a truly enjoyable read, and has been very well presented. I have really only one criticism and one request:Criticism:The Discussion gives a thoughtful analysis of the texts, but there is very little placement of the research work within the established literature. Granted, there is not as much literature on the topic as one would like (which is another reason supporting the value of this paper), but it does exist, and would serve as a stronger foundation for the research.Request:Normally, in qualitative research, one or two supporting quotes for each theme is sufficient. Given the novelty of the research, however, I really think it would be beneficial to the readers if the authors could supply more quotations. They may not wish to interrupt the flow of the paper, so a supplementary file with more quotes would help. (I do understand that the authors would probably prefer that the readers rather go to the periodical and read the original pieces, but that would not place the material within the context of the themes, so a middle-ground could be found with a supplementary file).One small error: “ever-increase amount”

Reviewer Expertise:

NA

No decision status is available

# Reviewer report: Reviewer response for version 1

**DOI:** 10.21956/mep.19957.r30319

**Published:** 2020-02-21

**Authors:** Barbara Jennings

**Affiliations:** 1 Norwich Medical School

This review has been migrated. The reviewer awarded 3 stars out of 5 I enjoyed reading this article, which describes a qualitative analysis of articles in a literary magazine that features writing and other works (e.g. artwork) from U.S. health care students.The rationale of the study (which was hard for me to grapple with at first read) was to explore the student’s insights about the necessary principles (or threshold concepts) associated with their professional development. The research goal was clearly presented “To understand the themes and perspectives of the published works”The authors describe the methods used fully; including each of their roles, the necessary research governance (appraisal by internal ethics review board), their coding strategy, and the thematic analysis & software used. Challenging aspects of objective coding are also described.The results describe thematic analysis of a subset of 130 pieces of published work, most of which were written pieces. In the discussion, the authors describe their aim to gain a “better understanding of the students’ perception of the medical profession”. They explore some fundamental challenges from the masks that professionals wear, to the ability to cope with ambiguity (as a critical threshold concept). I think there could have been a tighter link between the stated aim; methods; results and discussion. A lot of information seemed to be missing (for the reader) from the results section. For example, why was the information in the results section restricted to themes from the written work only? Could the authors consider adding a more comprehensive data set as supplementary information (this is possible for journals like MEP that have digital publications only)?The authors declare generalizability of their article as a possible limitation (given that editors and students are all from a single military medical school). However, I think this article will be of interest to teachers at most medical schools who consider the longitudinal, holistic development of medical students, particularly personal advisors and mentors.

Reviewer Expertise:

NA

No decision status is available
